# Simhypo-sand: a simple hypoplastic model for granular materials and SPH implementation

**DOI:** 10.1007/s11440-024-02350-8

**Published:** 2024-05-31

**Authors:** Shun Wang, Hong-jie Fang, Xuan Kang, Dian-qing Li, Wei Wu

**Affiliations:** 1 State Key Laboratory of Water Resources Engineering and Management, Wuhan University, Wuhan, China; 2 School of Water Resources and Hydropower Engineering, Institute of Engineering Risk and Disaster Prevention, Wuhan University, Wuhan, China; 3 Institute of Geotechnical Engineering, University of Natural Resources and Life Sciences, Vienna, Austria

**Keywords:** Critical state, Granular material, Hypoplastic model, Numerical simulation, Smoothed particle hydrodynamics

## Abstract

This paper introduces a new hypoplastic model characterized by a simple and elegant formulation. It requires only 7 material parameters to depict salient mechanical behaviors of granular materials. The numerical implementation employs an explicit integration method, enhanced by a best-fit stress correction algorithm in a smoothed particle hydrodynamics code. The performance of this model in capturing soil behavior across a range of scenarios is demonstrated by conducting various numerical tests, including triaxial and simple shear at low strain rates, as well as granular collapse, rigid penetration and landslide process at high strain rates.

## Introduction

Understanding and modeling of the mechanical response of granular materials have been the focus of numerous studies, primarily because granular materials exhibit diverse mechanical behaviors stemming from variations in their densities. As it is widely acknowledged, dense sand displays an initial contractancy followed by subsequent dilatancy; whereas, loose sand consistently remains in a condensed state. Much like clay, the critical states play a pivotal role in determining the ultimate deformation and stress conditions of granular materials [33]. To understand the complex behaviors of granular materials, numerous constitutive models based on the theory of elastoplasticity have been developed over the past few decades. Noteworthy among these are kinematic hardening plasticity [38], bounding surface plasticity [8], and generalized plasticity [28]. Recent models have also incorporated the concept of the critical state [44, 45]. These models have consistently demonstrated exceptional performance in predicting the mechanical behaviors of soils.

Hypoplasticity is an alternative approach for modeling mechanical behaviors of soils. In contrast to traditional elastoplastic constitutive models, hypoplasticity offers the capability to describe anelastic phenomena without the need for additional concepts such as yield surfaces, plastic potentials, and strain decomposition. These concepts are inherent byproducts of the constitutive equations [46]. Hypoplastic models demonstrate predictive capabilities that stand on par with those of advanced models in elastoplastic frameworks. Despite their simplicity, early versions of hypoplastic models [17, 37, 46] are able to reasonably capture some salient features of granular materials, such as sands and gravels with only four parameters. Later, an important step forward was taken by incorporating the critical state concept. For example, Wu et al. [47] enchanced the original four-parameter hypoplastic model [46] with a critical state function that considers the effects of density variations using a straightforward formulation. In 2017, Wu et al. [48] introduced a basic four-parameter hypoplastic model for sand. This model is practical because it requires only four parameters, making it simpler than many other elastoplastic models. Subsequent versions of hypoplastic models greatly expand their capabilities for predicting various soil features [10, 11, 20–22, 40, 41]. Moreover, Wang et al. [42] implemented the basic four-parameter model within the framework of finite element method (FEM) to solve boundary value problems in geotechnical practices. Enhanced by the availability of robust algorithms for seamless integration into numerical codes, this development makes hypoplasticity a promising approach for practical applications. Besides the simplicity of hypoplastic model, the rate form of the constitutive equations is also a desirable feature for solving large deformation problems with advanced numerical methods, such as smoothed particle hydrodynamics (SPH).

The implementation and application of hypoplasticity in SPH simulation have gained increasingly popularity in recent year. The first attempts for implementation hypoplasticity into the SPH methodology was carried out by Peng et al. [29–31], who implemented the four-parameter basic hypoplastic model by Wu et al. [48] into an in-house SPH code. This effort demonstrated that the efficacy of hypoplastic model in simulating geotechnical problems and granular flows involving large deformation. Later, Chen et al. [6] adopted the original four-parameter original hypoplastic model [46] for landmine detonation in SPH simulation. This model was further validated in SPH simulation of pile installation [36]. Zhao et al. [52] implemented the hypoplastic model by Gudehus and Bauer [2, 9] into SPH for simulating granular biomass. More recently, Zhu et al. [53] implemented the four-parameter basic hypoplastic model into SPH simulation for granular materials in large deformation problems. These advancements collectively underscore the growing significance of hypoplasticity within the SPH framework for addressing complex problems in various fields.

While hypoplasticity is commonly applied in geotechnical simulations, achieving a constitutive-based solid mechanics calculation within the pure Lagrangian framework, especially in methods like SPH, presents a significant challenge. This is because, in conventional SPH method, pressure (stress) is computed from density variations using an equation of state [25, 51]. Although this approach works well for fluids, it is evidently oversimplified for geomaterials. Some simplified models, such as depth integration methods [27] and the Bingham rheology [54], which are based on fluid mechanics, are inadequate to reproduce the mechanical behaviors of granular materials. Moreover, in simulations involving large deformations, materials often exhibit substantial changes in their kinematics, deviating significantly from experimental observations. This situation generates numerical instability, which significantly restricts the practical application of the hypoplastic theory [16]. It underscores the importance of developing a stable and robust model that shows both simple expression and accurate prediction for granular materials. In this paper, a new critical state-based hypoplastic constitutive model named as simhypo-sand model is introduced. Only 7 parameters are required to capture the salient behaviors of the granular materials considering variation of void ratio. This model incorporates a controllable failure surface that varies between the Drucker–Prager and spatially mobilized plane (SMP) criteria. To ensure numerical stability in simulating large deformation problems, a stress correction scheme is proposed to regulate stress states prone to pathologies.

The remaining content of this paper is organized as follows: First, the fundamental equations of the proposed simplified hypoplastic model are presented in Sect. 2. This includes deriving the explicit expression of its failure surface and explaining the physical meanings of its parameters. Then, the numerical framework of the hypoplastic model within SPH framework is illustrated, which encompasses the procedures for conducting integration schemes, and implementing stress correction algorithms. In what follows, an evaluation is conducted on the ability of the proposed model to depict the constitutive relationship of granular material through a series of elementary tests. Furthermore, in Sect. 5, the practical applicability of this model is validated by carrying out classical benchmark problems involving large deformation, such as granular collapse, penetrator in sand, and landslide deposition.

*Notation and conventions*: Second-order tensors are denoted with bold letters (e.g., $\varvec{\sigma }$, $\varvec{\epsilon }$). The tensor multiplication rules are: $\textbf{A}\cdot\textbf{B}$ = $A_{ik}B_{kj}$ and **A**:**B** = $A_{ij}B_{ij}$. The quantity $\Vert {\textbf {A}}\Vert$ = $\sqrt{{\textbf {A}}:\textbf {A}}$ denotes the Euclidean norm of **A**. tr(**A**)= ${\textbf {A}}_{ii}$ refers the trace of **A**, and ${\textbf {A}}^{*}$ = **A**-tr(**A**)**I**/3 signifies the deviatoric part of **A** with **I** being the second-order unity tensor. In the following, except where specified otherwise, effective stress is used.

## Theory of hypoplastic constitutive model

### General framework

Generally, a hypoplastic constitutive equation describes the evolution of stress by adopting a nonlinear tensorial function, which is consisted of a linear and a nonlinear parts, representing the reversible and irreversible behavior of granular materials, respectively. The following framework proposed by Wu et al. [47] is the first attempt to consider the critical state of granular materials in a hypoplastic model:

1 $$\begin{aligned} \mathring{\varvec{\sigma }}=f_s\left[ \varvec{\mathcal {L}}(\varvec{\sigma },\dot{\varvec{\epsilon }})+f_d{\textbf {N}}(\varvec{\sigma },\dot{\varvec{\epsilon }})\right] \end{aligned}$$

where $\varvec{\sigma }$ is the Cauchy stress; $\mathring{\varvec{\sigma }}$ is the Jaumann stress rate, and $\dot{\varvec{\epsilon }}$ is the strain rate (stretching) tensor. $\varvec{\mathcal {L}}$ and ${\textbf {N}}$ are fourth- and second-order tensorial function, which are assumed to be linear and nonlinear in the stretching tensor, respectively. $f_s$ and $f_d$ are the stiffness and density factors accounting for the effects of material stiffness and void ratio (*e*) during loading in the granular materials, respectively.

The Jaumann rate of the Cauchy stress tensor $\mathring{\varvec{\sigma }}$ is defined in terms of the time derivative of the Cauchy stress tensor $\dot{\varvec{\sigma }}$ and the spin tensor $\varvec{w}$, as follows:

2 $$\begin{aligned} \mathring{\varvec{\sigma }}=\dot{\varvec{\sigma }}+\varvec{\sigma } \varvec{w}-\varvec{w} \varvec{\sigma } \end{aligned}$$

The above stretching and spin tensors are related to the velocity gradient tensor through

3 $$\begin{aligned} \dot{\varvec{\epsilon }}=\frac{1}{2}(\varvec{\nabla v}+\varvec{ v \nabla }),\quad \varvec{w}=\frac{1}{2}(\varvec{\nabla v}-\varvec{ v \nabla }) \end{aligned}$$

where $\varvec{v}$ is the velocity and $\varvec{\nabla }$ is the gradient operator.

### A critical state hypoplastic model for sand

According to the basic hypoplastic by Wu et al. [48], a simple hypoplastic constitutive equation is proposed to describe the mechanical behavior of granular materials:

4 $$\begin{aligned} \mathring{\varvec{\sigma }}=f_s\Bigg[\textrm{tr}(\varvec{\sigma })\dot{\varvec{\epsilon }}+ f_v\textrm{tr}(\dot{\varvec{\epsilon }})\varvec{\sigma }+ a^2\frac{\textrm{tr}(\varvec{\sigma }\dot{\varvec{\epsilon }})}{\textrm{tr}(\varvec{\sigma })}\varvec{\sigma }+ f_da(\varvec{\sigma }+\varvec{\sigma }^{*})\Vert \dot{\varvec{\epsilon }}\Vert \Bigg] \end{aligned}$$

where $f_v$ is a multiplier related to the volumetric responses of a granular material; *a* is a material constant to describe the shear strength at critical state. $f_d$ is the density factor, which incorporates the critical state of granular materials in the following way:

5 $$\begin{aligned} f_d=\left( \frac{e}{e_c}\right) ^\alpha \end{aligned}$$

in which *e* is the current void ratio; $e_c$ is the critical state void ratio with the subscript *c* denoting the critical state. The value of $f_d$ is less than 1 for a dense state, greater than 1 for a loose state, and equals 1 at the critical state. The parameter $\alpha$ regulated the effect $f_d$ on the constitutive response [43].

The evolution of critical state follows a linear model proposed by Li and Wang [19] since it has been widely used to describe the critical state line of granular materials in the compression plane. Accordingly, the critical state void ratio is determined as follows:

6 $$\begin{aligned} e_c=e_\Gamma -\lambda \left( \frac{p^\prime }{p_\text {a}}\right) ^\xi \end{aligned}$$

where $e_\Gamma$, $\lambda$, and $\xi$ are model parameters for characterizing the critical state of granular materials. $p^\prime =-\text {tr}(\varvec{\sigma })/3$ is the effective mean stress and $p_\text {a}$ = 101.325 kPa is the atmospheric pressure. The normalization of $p^\prime$ with respect to $p_\text {a}$ in Eq. (6) makes the intercept parameter $\lambda$ independent of the unit of stress.

### Calibration of $f_s$ and $f_v$

The multipliers $f_s$ and $f_v$ can be calibrated from a single drained triaxial compression test. To do this, the equation of model (4) can be decomposed into the hydrostatic ($\dot{p}$) and deviatoric ($\dot{q}$) parts, as follows:

7a $$\begin{aligned} \dot{p}= & {} pf_s \Biggl [ \Bigl ( 1+ \frac{a^2}{3}+f_v \Bigr ) \dot{\epsilon }_v + a^2 \hat{q}\dot{\epsilon }_q + f_da\sqrt{ \frac{\dot{\epsilon }_v^2}{3}+\frac{3}{2}\dot{\epsilon }_q^2} \, \Biggr ] \end{aligned}$$

7b $$\begin{aligned} \dot{q}= & {} -3pf_s \Biggl [ \frac{3}{2}\dot{\epsilon }_q + \bigg ( \frac{a^2}{3}+f_v\bigg )\dot{\epsilon }_v\hat{q} + a^2\dot{\epsilon }_q \hat{q}^2 + 2f_d\hat{q}a\sqrt{\frac{\dot{\epsilon }_v^2}{3}+\frac{3}{2}\dot{\epsilon }_q^2} \, \Biggr ] \end{aligned}$$

$\dot{\epsilon }_v = \textrm{tr}(\dot{\varvec{\epsilon }})$ and $\dot{\epsilon }_q=-\sqrt{2/3}\Vert \dot{\varvec{\epsilon }}^* \Vert$ stand for the volumetric and deviatoric strain rates, respectively; $\hat{q}=q/\text {tr}(\varvec{\sigma })$ is the normalized deviatoric stress.

Next, we consider a drained triaxial compression test with a constant confining pressure $\sigma _c$ and an axial strain rate $\dot{\epsilon }_{a}$. Initially, the deviatoric stress $q_i=0$, and the radial strain rate can be obtained by $\dot{\epsilon }_{ri} =-\nu _i \dot{\epsilon }_{a}$ with $\nu _i$ being the initial Poisson’s ratio. Therefore, we have the following initial condition:

8 $$\begin{aligned} \dot{\epsilon }_v=\dot{\epsilon }_{a}(1-2\nu _i),\; \dot{\epsilon }_q=\frac{2}{3}\dot{\epsilon }_{a}(1+\nu _i),\; \hat{q}=0 \end{aligned}$$

Substituting the above initial condition into Eq. (7) yields the following initial stress components:

9a $$\begin{aligned}&\dot{p}_i=\sigma _cf_s \dot{\epsilon }_{a} \bigg [ \left(1+\frac{1}{3} a^2 +f_v\right) (1-2\nu _i) -f_d a\sqrt{1+2\nu _i^2} \, \bigg ] \end{aligned}$$

9b $$\begin{aligned}&\dot{q}_i = -3\sigma _cf_s\dot{\epsilon }_{a} (1+\nu _i) \end{aligned}$$

On one hand, the drained triaxial compression test is characterized by the stress path ratio $\dot{q}/\dot{p}=-3$; and thus, the multiplier $f_v$ is obtained as:

10 $$\begin{aligned} f_v= \frac{3\nu _i+f_da\sqrt{1+2\nu _i^2}}{1-2\nu _i} -\frac{a^2}{3} \end{aligned}$$

On the other hand, the deviatoric stress rate is related to the strain rate through the initial shear modulus, i.e., $E_i=\dot{q}_i / \dot{\epsilon }_{a}$. Therefore, the stiffness factor can be obtained from Eq. (9b):

11 $$\begin{aligned} f_s= -\frac{E_i}{3(1+\nu _i)\sigma _c} \end{aligned}$$

in which a confining pressure of $\sigma _c=100$ kPa is usually adopted for parameter calibration.

### Failure surface

To derive the failure surface of model (4), we can write Eq. (1) in the following form with respect to the Euler’s theorem for homogeneous functions.

12 $$\begin{aligned} \mathring{\varvec{\sigma }}=f_s[\varvec{\mathcal {L}}+f_d{\textbf {N}}\otimes \vec {\dot{\varvec{\epsilon }}}]:\dot{\varvec{\epsilon }} \end{aligned}$$

where $\vec {\dot{\varvec{\epsilon }}}=\dot{\varvec{\epsilon }}/\Vert \dot{\varvec{\epsilon }}\Vert$ is the direction of strain rate. The term in the square brackets in Eq. (12) represents the directional stiffness tensor, which is incrementally nonlinear, i.e., dependent on the direction of strain rate $\vec {\dot{\varvec{\epsilon }}}$. Equation (12) describes a critical state condition for continuing deformation when the directional stiffness vanishes, then we have $\mathring{\varvec{\sigma }}=0$, which corresponds to

13 $$\begin{aligned} -f_d\varvec{\mathcal {L}}^{-1}:{\textbf {N}}=\vec {\dot{\varvec{\epsilon }}} \end{aligned}$$

The above equation represents the flow rule of the hypoplastic model. For the sake of simplicity, let’s assume a tensorial function ${\textbf {B}}=-\varvec{\mathcal {L}}^{-1}:{\textbf {N}}$. Since $\Vert \vec {\dot{\varvec{\epsilon }}} \Vert =1$, taking the norm of Eq. (13) gives rise to the failure criterion:

14 $$\begin{aligned} f(e,\varvec{\sigma })= f_d\Vert {\textbf {B}}\Vert -1=0 \end{aligned}$$

**Fig. 1:**
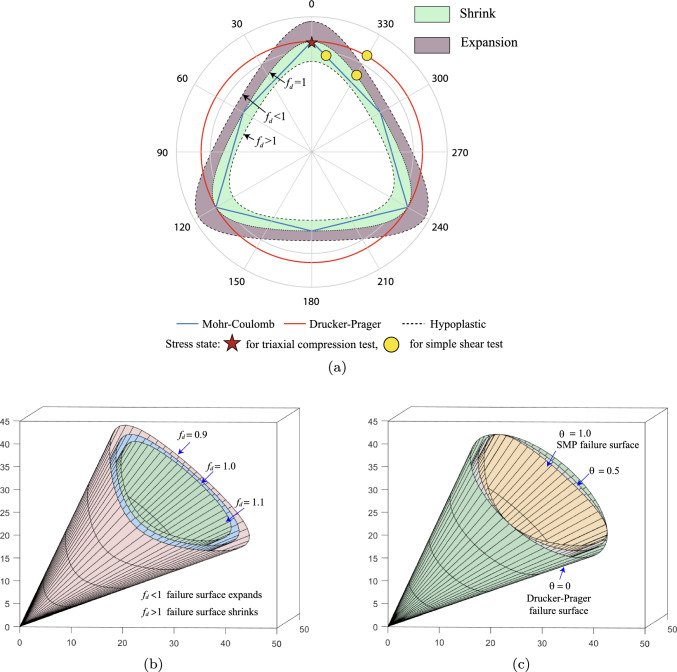
Features of hypoplastic failure surface with $\phi _c$=30^∘^ **a** comparison with Mohr–Coulomb and Drucker–Prager failure surfaces in $\pi$ plane **b** evolution of hypoplastic failure surface under the influence of $f_d$ **c** transition from Drucker–Prager to SMP failure surface

The tensorial function ${\textbf {B}}$, which defines the flow rule of a hypoplastic model, has been used in the development of several hypoplastic constitutive models [24, 49]. For model (4), the explicit formulation of ${\textbf {B}}$ is expressed as follows:

15 $$\begin{aligned} {\textbf {B}}=\frac{a}{\textrm{tr}(\varvec{\sigma })} \Bigg [ \varvec{\sigma }^*+ \varvec{\sigma } \frac{\textrm{tr}(\varvec{\sigma })^2- a^2 \Vert \varvec{\sigma }^* \Vert ^2 }{(1+f_v) \textrm{tr}(\varvec{\sigma })^2+ a^2 \Vert \varvec{\sigma }\Vert ^2 } \Bigg ] \end{aligned}$$

The parameter *a* in Eq.(15) is equal to the normalized deviatoric stress $\Vert \varvec{\sigma }_c^* \Vert /\textrm{tr}(\varvec{\sigma }_c)$ at critical state, giving

16 $$\begin{aligned} a_c = \frac{\sqrt{3}(3-\text {sin}\phi _c)}{2\sqrt{2}\text {sin}\phi _c} \end{aligned}$$

in which $\phi _c$ is the critical state friction angle. With a constant value for *a*, the failure surface has a conical shape in the principal stress space, which resembles that of the Drucker–Prager failure surface [48]. In order to describe the soil behavior under multiaxial conditions, a more promoting and flexible strength criterion proposed by Yao et al. [50] is incorporated in this model. To the end, the following formulation is adopted for *a*:

17 $$\begin{aligned} a=\frac{a_c}{\theta q_m/q_s+1-\theta } \end{aligned}$$

where $\theta$ is a weighted factor ranging between 0 and 1, with the lower and upper bounds corresponding to the Drucker–Prager and SMP criteria. $q_m$ and $q_s$ are the corresponding radius of failure surface on the deviatoric planes under triaxial compression stress state for the Drucker–Prager criterion and the SMP criterion, respectively, with

18 $$\begin{aligned} q_m= \sqrt{I_1^2-3I_2},\; q_s= \frac{2I_1}{3\sqrt{(I_1I_2-I_3)/(I_1I_2-9I_3)-1}} \end{aligned}$$

in which $I_1$, $I_2$, and $I_3$ are stress invariants.

A significant feature of the proposed hypoplastic model is that it can consider the influence of void ratio on the stress–strain relationship of granular material. Through the parameter $f_d$, the shape of the failure surface contracts or expands with changes in void ratio (as illustrated in Fig. 1a and b), thus simulating the stress–strain behavior of sandy soils under different degrees of compaction. When the void ratio is less than the critical state void ratio ($f_d < 1$), dense sand exhibits noticeable strain softening. Conversely, loose sand ($f_d > 1$) displays strain hardening. In contrast, traditional models that do not account for density variation may fail to accurately replicate the observed behavior, highlighting the advantage of the proposed model in simulating the deformation behavior of granular materials. Therefore, the density factor plays a similar role of a hardening law resembling that used in an elastoplastic constitutive model. In addition, by adjusting the weight value $\theta$ between 0 and 1, a smooth transition of the failure criterion from Drucker–Prager to SMP type can be achieved, as shown in Fig. 1c. In this work, the SMP failure criterion with $\theta =1$ is adopted for granular materials. Appendix A provides an overview of the simhypo-sand model, including the methods for determining material parameters.

## Numerical implementation in SPH

### Governing equations

The governing equations of soil in framework of SPH consist of mass and momentum conservation equations as follow:

19 $$\begin{aligned} \frac{\textrm{d}\rho }{\textrm{d}t}= & {} -\rho \nabla \cdot \varvec{v} \end{aligned}$$

20 $$\begin{aligned} \frac{\textrm{d}\varvec{v}}{\textrm{d}t}= & {} \frac{1}{\rho }\nabla \cdot \varvec{\sigma } + \varvec{g} \end{aligned}$$

where $\rho$ is the density; $\varvec{v}$ denotes the velocity; $\varvec{\sigma }$ is the Cauchy stress tensor, and $\varvec{g}$ is the acceleration induced by body force, e.g., gravity in most geotechnical problems. $\textrm{d}(\cdot )/\textrm{d}t$ represents the material time derivative and $\nabla$ is the gradient operator. In this work, pore pressure is not considered, which means the material is either dry or can be modeled using total stress.

As discussed by Peng et al. [29], the use of the continuity equation in the current SPH application for hypoplastic models is optional. Provided that the soil density remains constant, the continuity equation can be omitted from the governing equations. In SPH, the problem domain is composed of particles, and all parameters within the domain can be interpolated as sums of contributions from the particles in the support domain. In this context, we follow the discrete forms of SPH interpolation.

21 $$\begin{aligned} f(\varvec{x}_{i})= & {} \sum _{j=1}^{n}f( \varvec{x}_{j})W_{ij}m_{j}/\rho _j \end{aligned}$$

22 $$\begin{aligned} \nabla f(\varvec{x}_{i})= & {} \sum _{j=1}^{n} f(\varvec{x}_{j}) \nabla _i W_{ij}{m_{j}}/{\rho _j} \end{aligned}$$

where *n* is the number of particles within the support domain, the subscripts $\varvec{x}_{i}$ and $\varvec{x}_{j}$ denote the evaluated particle and the neighboring particles within the support domain; $\nabla _i$ indicates the derivatices are evaluated at particle *i*; $m_j$ and $\rho _j$ are the mass and density of particle *j*, respectively; $W_{ij}=W(\varvec{x}_{i}-\varvec{x}_{j},h)$ represents the kernel function with *h* being the smoothing length. This study adopted the Wendland C2 kernel due to its ability to mitigate the clumping of particles [31]:

23 $$\begin{aligned} W=\alpha _d {\left\{ \begin{array}{ll} (1-0.5R)^4(2R+1) &{}\text {if } 0\le R \le 2, \\ 0 &{} \text {if } R>2. \end{array}\right. } \end{aligned}$$

in which *R* is the normalized distance between two particles and defined as $R = r/h$ with *r* and *h* being the distance between two particles and the smooth length, respectively; $\alpha _d$ is a normalization factor, and the value is $7/(16\pi h^2)$ in two dimensions and $21/(64\pi h^3)$ in three dimensions.

By applying the aforementioned procedure of particle approximation, the following expressions are adopted to discretize the governing equations:

24 $$\begin{aligned} \frac{\textrm{d}\rho _i}{\textrm{d}t}= & {} \sum _{j=1}^{n}m_j(\varvec{v}_i-\varvec{v}_j)\cdot \nabla _i W_{ij} \end{aligned}$$

25 $$\begin{aligned} \frac{\textrm{d}\varvec{v}_i}{\textrm{d}t}= & {} \sum _{j=1}^{n}m_j\left( \frac{\varvec{\sigma }_i}{\rho _i^2}+\frac{\varvec{\sigma }_j}{\rho _j^2}+\Pi _{ij}\varvec{\varvec{I}}\right) \nabla _i W_{ij}+\varvec{g}_i \end{aligned}$$

where $\varvec{I}$ represents the identity matrix; the dissipative term $\Pi$ is the artificial viscosity, which enhances the numerical stability and prevents large unphysical oscillations, reading

26 $$\begin{aligned} \Pi _{ij} = {\left\{ \begin{array}{ll} \dfrac{-\alpha _\Pi c_s \phi _{ij} + \beta _\Pi \phi _{ij}^2}{\overline{\rho _{ij}}}, &{} \text {if } \varvec{v}_{ij} \cdot \varvec{r}_{ij} < 0, \\ 0, &{} \text {otherwise}. \end{array}\right. } \end{aligned}$$

with

27 $$\begin{aligned} \phi _{ij} = \frac{h\varvec{v_{ij}}\cdot \varvec{r}_{ij}}{\Vert \varvec{r_{ij}}\Vert ^2+0.01h^2},\ \text {and}\quad \overline{\rho _{ij}}= \frac{\rho _i+\rho _j}{2} \end{aligned}$$

where $\varvec{v}_{ij} = \varvec{v}_i - \varvec{v}_j$ and $\varvec{r}_{ij} = \varvec{r}_i - \varvec{r}_j$; $c_s$ is the speed of sound in soil; $\alpha _\Pi$ and $\beta _\Pi$ are constants determining the magnitude of artificial viscosity. The appropriate values of $\alpha _\Pi$ and $\beta _\Pi$ should be tuned in particular problem. Given that the primary focus of this study is non-cohesive sand, tensile instability is generally negligible. However, in situations where the simulation involves materials subjected to tensile stresses, addressing the issue of tensile instability becomes crucial. One effective approach to manage this challenge is through the application of artificial stress. Further details on this treatment can refer to the works by Bui et al. [5] and Peng et al. [31].

### Explicit time integration

Numerous conventional numerical techniques exist for computing discrete SPH equations, including the prediction–correction method [31], the second-order leapfrog method, and the Runge–Kutta scheme [5]. In the present study, the Verlet scheme [29] has been adopted for subsequent simulations. The update equations for the variables are delineated as follows:

28 $$\begin{aligned} \varvec{v}_i^{n+1}=\varvec{v}_i^{n-1}+2\Delta t\frac{d\varvec{v}_i^n}{dt} \end{aligned}$$

29 $$\begin{aligned} \varvec{x}_i^{n+1}=\varvec{x}_i^{n}+\Delta t\varvec{v}_i^{n}+0.5\Delta t^2 \frac{d\varvec{v}_i^n}{dt} \end{aligned}$$

30 $$\begin{aligned} \varvec{\sigma }_i^{n+1}=\varvec{\sigma }_i^{n-1}+2\Delta t\frac{d\varvec{\sigma }_i^n}{dt} \end{aligned}$$

31 $$\begin{aligned} \varvec{\epsilon }_i^{n+1}=\varvec{\epsilon }_i^{n-1}+2\Delta t\frac{d\varvec{\epsilon }_i^n}{dt} \end{aligned}$$

32 $$\begin{aligned} e_i^{n+1}= e_i^{n-1}+2\Delta t (1+e_i^{n})\textrm{tr}(\dot{\varvec{\epsilon }}_i^n) \end{aligned}$$

where $n-1,n,n+1$ denote the time steps.

To guarantee the stability of above Verlet scheme, a variable time step $\Delta t$ is determined using Courant–Friedrichs–Lewy (CFL) condition and the acceleration term:

33 $$\begin{aligned} \Delta t_1=\mathop {min}\limits _{i}(\sqrt{h/\Vert \varvec{a}_i\Vert }) \end{aligned}$$

34 $$\begin{aligned} \Delta t=\chi _\text{CFL}\mathop {min}(\Delta t_1, h/c_s) \end{aligned}$$

where $\varvec{a}_i$ is the acceleration of particle *i*; $\chi _\text{CFL}$ is the CFL coefficient; and $c_s$ is the artificial speed of sound. The $c_s$ is determined by the shear velocity of particle and $\chi _\text{CFL}$ is set as 0.05, which provides a small time step for the computation stability.

Note that the stress rate in Eq. (30) is updated with the hypoplastic constitutive model. Since this model is in rate form, its discretization in SPH is quite straightforward. For a given particle *i* the stress rate can be written as:

35 $$\begin{aligned}{} & {} \frac{d\varvec{\sigma }_i}{dt}= \varvec{w}_i \varvec{\sigma }_i -\varvec{\sigma }_i\varvec{w}_i +f_s\Big [\textrm{tr}(\varvec{\sigma }_i)\dot{\varvec{\epsilon }}_i+ f_v\textrm{tr}(\dot{\varvec{\epsilon }}_i)\varvec{\sigma }_i\\ & \quad + a^2\frac{\textrm{tr}(\varvec{\sigma }_i\dot{\varvec{\epsilon }}_i)}{\textrm{tr}(\varvec{\sigma }_i)}\varvec{\sigma }_i+ f_da(\varvec{\sigma }_i+\varvec{\sigma }^{*}_i)\Vert \dot{\varvec{\epsilon }}_i\Vert \Big ] \end{aligned}$$

where $\varvec{w}_i$, $\varvec{\sigma }_i$, $\dot{\varvec{\epsilon }}_i$ are the spin tensor, stress tensor, and strain rate tensor at particle *i*, respectively. The spin tensor and strain rate at particle *i* in Eq. (35) are computed from the velocity gradient as given by Eq. (3) and its SPH approximation over particles in the support domain is obtained by applying Eq. (22). The stress rate tensor is calculated directly from the stress state and strain rate through the above constitutive model without splitting strain tensor into elastic and plastic parts as elastoplasticity always does.

It is widely acknowledged that the intricate process of stress return mapping, typically imperative in elastoplastic models, is dispensable for hypoplastic models. This holds true under conditions where the velocity field exhibits regularity, as observed in elementary tests. However, the numerical computation becomes notably precarious in boundary value problems characterized by dynamic kinematics with substantial variations, such as in granular flows. The challenge arises from the random encounters of SPH particles with rapid movement, leading to elevated strain rates, especially in proximity to the free surface. Consequently, at the conclusion of a finite calculation step, the stress state may deviate significantly from the failure surface–a deviation incompatible with plasticity theory. If left unaddressed, this discrepancy in the stress state could propagate unreasonably in subsequent steps, exacerbating unphysical kinematics and potentially resulting in the collapse of the calculation. Therefore, the implementation of a judicious scheme to regulate the stress state becomes imperative.

### Stress correction algorithm

At the end of each time step in the integration process, the stresses inevitably diverge from the failure condition, leading to $f(\varvec{\sigma },e)>0$. The extent of this violation, which is commonly known as ‘stress drift’, depends on the accuracy of the integration scheme and the nonlinearity of the constitutive relations. Sloan [35] suggests that, provided the integration is performed accurately, the extent of drift from the failure surface will tend to be small and no remedial action is required. However, the fact is that the hypoplastic model (4) describes a strongly nonlinear relationship between stress rate and state-dependent variables, i.e., strain rate and void ratio. The effect of stress drift is cumulative and may lead to numerical instability once the stress error surpass a threshold. Therefore, numerical integration of a hypoplastic model requires a very high accuracy, which can only be achieved by adopting some forms of stress correction in the explicit integration scheme.

**Fig. 2:**
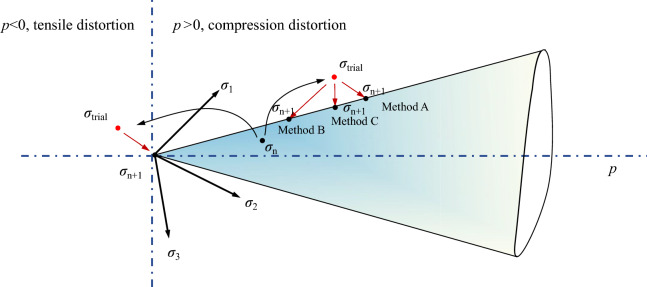
Illustration of stress correction algorithm

Figure 2 illustrates potential unrealistic stress states that soil particles may experience during the integration process, including tensile and compressive distortions. In the integration process from step *n* to $n+1$, the procedure commences by evaluating the need for stress correction based on a trial stress ($\sigma _{\text {trial}}$). If the trial stress exceeds the failure surface and exhibits tensile distortion, the stress state should be repositioned to the origin of the coordinate system. In the case of stress states displaying compressive distortion, a rollback toward the failure surface should be executed in the appropriate direction. In this study, three stress correction algorithms, namely Method A (projecting back along the flow rule), Method B (projecting back along the stress rate direction), and Method C (projecting back by reducing $J_2$ at constant $I_{1}$) are utilized. This method was initially introduced by Potts and Gens [32] in an elastoplastic model and subsequently pioneered its application for implementing hypoplasticity in FEM by Wang et al. [42]. The expressions for Method A and Method B are shown as follows:

36 $$\begin{aligned} \text {Method A: }\varvec{\sigma }_\text{correction}=\varvec{\sigma }_\text{trial}-\alpha _{A}{} {\textbf {B}},\quad \alpha _{A}=\frac{f(e,\varvec{\sigma })}{({\partial f}/{\partial \varvec{\sigma }}) {\textbf {B}}} \end{aligned}$$

37 $$\begin{aligned} \text {Method B: }\varvec{\sigma }_\text{correction} = \varvec{\sigma }_\text{trial} - \alpha _{B}\dot{\varvec{\sigma }},\quad \alpha _{B} = \frac{f(e,\dot{\varvec{\sigma }})}{({\partial f}/{\partial \varvec{\sigma }})\dot{\varvec{\sigma }}} \end{aligned}$$

where $\alpha _{A}$ and $\alpha _{B}$ denote the correction factor; **B** defines the flow rule expressed in Eq. (15). For Method A, the correction is implemented along the flow rule; while for Method B, the correction is implemented along the stress rate direction. As for Method C, the expression is delineated as follows:

38 $$\begin{aligned} \text {Method C:} \quad \varvec{\sigma }_{\text {correction}}^{*}=\alpha _{c}\varvec{\sigma }_\text {trial}^{*},\quad I_{1(\text {correction})}=I_{1(\text {trial})} \end{aligned}$$

where $\alpha _{c}$ serves as the correction factor adjusting the trial values of $J_2$ and $I_1$. Due to the variable failure surface inherent in the proposed hypoplastic model, $\alpha _{c}$ cannot typically be explicitly executed. In general scenarios, the implementation of Method C can be realized through numerical algorithms, such as the Newton–Raphson method or Binary Search. This involves projecting back by reducing $J_2$ while keeping $I_{1}$ constant, relying on the explicit formulation of the failure function detailed in Eq. (13). If the Drucker–Prager failure surface is utilized, a corresponding stress correction scheme can be explicitly formulated, as exemplified in Appendix B.

**Fig. 3:**
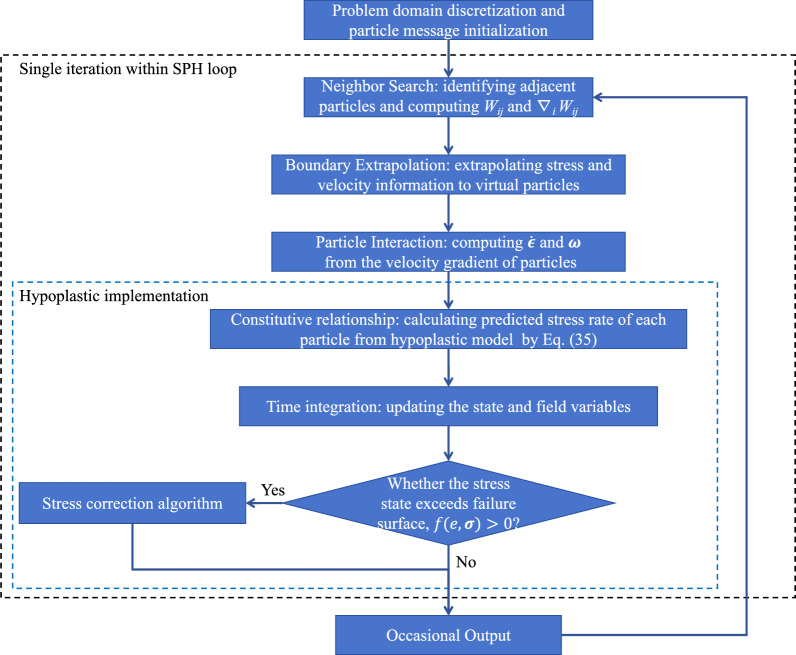
Flowchart of SPH simulation process

### Implementation in SPH

To elucidate the implementation of the hypoplastic model in SPH, we have synthesized the aforementioned procedures into a comprehensive framework, as illustrated in Fig. 3. The main steps can be outlined as follows:

1. Initialization: firstly, the problem domain is discretized into a finite set of particles, and each particle is assigned initial values for material properties and state variables. Neighborhood search is performed based on particle positions to determine contact relationships between particles, and to compute the values of the kernel function $W_{ij}$ and its gradient $\nabla _i W_{ij}$.
2. Boundary treatment: Based on the results of the neighborhood search, velocities, stresses, and other state variables are assigned to boundary particles by the kernel function $W_{ij}$ using the method proposed by Bui et al. [5].
3. Hypoplastic model application: Following the calculation of velocity gradient differences between particles in the current step, the strain rate and spin tensor for each particle are determined. The proposed hypoplastic model Eq. (35) is directly applied to compute the particle’s stress rate.
4. Time integration and stress correction: Finally, the trial results for the next time step are obtained through time integration. A check is performed on the stress state, evaluating the value of the failure function. If it exceeds 0, indicating that the particle’s stress has surpassed the yield surface, corrective measures are implemented. The information for each particle is updated using the corrected results, serving as either the output or the initial information for the subsequent computational step.

## Numerical simulation of elementary tests

To give an overall assessment of the performance of the proposed model and adopted stress correction algorithm, a set of elementary tests, including drained triaxial compression tests and simple shear tests, are carried out using a MATLAB script. The numerical results of the model and two conventional elastoplastic models, the Mohr–Coulomb and the Drucker–Prager, are compared. The parameters listed in Table 1 are used in the simulations.

**Table 1:** Material parameters for simulating triaxial drained and simple shear tests

Mohr–Coulomb		Drucker–Prager		Hypoplasticity			
Parameter	Value	Parameter	Value	Parameter	Value	Parameter	Value
*E*	12.0 MPa	*E*	12 MPa	$E_i$	12 MPa	$\xi$	0.2
$\nu$	0.15	$\nu$	0.15	$\nu _i$	0.15	$\alpha$	1.8
$\phi _c$	30.0^∘^	$\phi _c$	30.0^∘^	$\phi _c$	30.0^∘^	$e_0$	0.98$-$1.18
$\psi$	0.10	$\psi$	0.10	$\lambda$	0.126	$e_{\Gamma }$	1.245

*E*, $\nu$ and $\psi$ are the elastic modulus, Poisson’s ratio and dilatancy angle, respectively

### Drained triaxial compression test

**Fig. 4:**
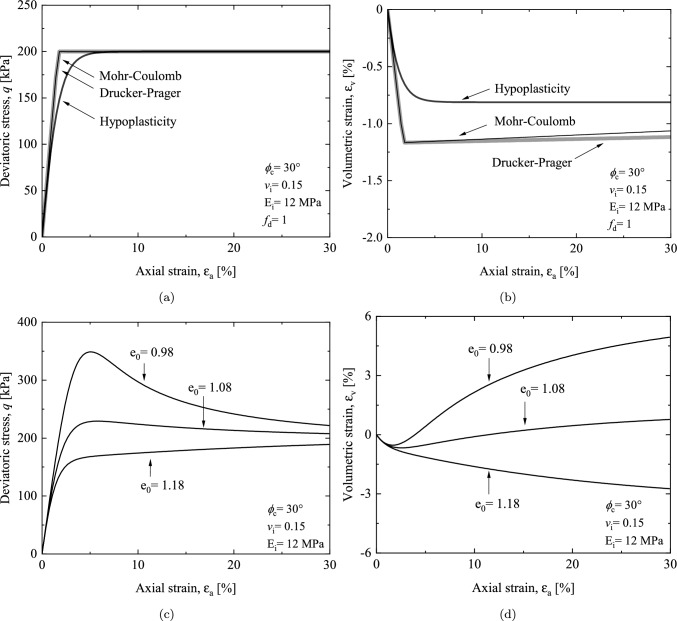
Numerical simulation of drained triaxial compression tests with $\sigma _3$= 100 kPa: **a**,**b** axial strain–stress relation and axial strain–volumetric strain using different constitutive models, **c**,**d** axial strain–stress relation and axial strain–volumetric strain with various void ratio

The numerical test starts from a hydrostatic stress state with the constant confining pressure being 100 kPa and an initial void ratio of 1.08. It can be observed from Fig. 4a that the numerical tests give rise to the same failure deviatoric stress regardless of the model used; while, the test using the hypoplastic model gains a nonlinear strain–stress response. The results shown in Fig. 4b reveals that different models result in similar volumetric responses and all the three models give contractive volume changes.

Figures 4c and 4d demonstrate the performance of the proposed hypoplastic model in depicting the critical state of granular materials under varying compaction levels. The same initial confining pressure of 100 kPa is used in this simulation. As the compaction level increases, there is a corresponding rise in the peak shear strength. This transformation in strain–stress behavior is accompanied by a change in the soil’s volumetric strain response. Initially characterized by shear contraction, then the behavior subsequently transitions toward shear dilation before the critical state is achieved.

**Fig. 5:**
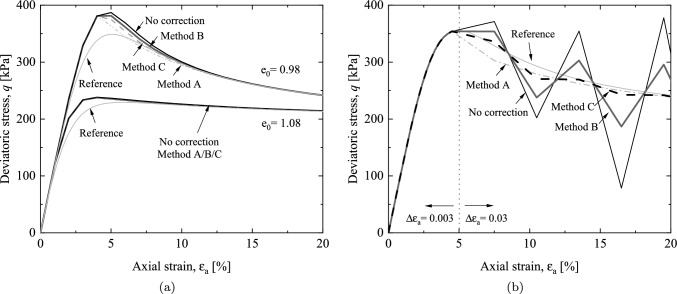
Performance of stress correction algorithm in drained triaxial compression tests with $\sigma _3$= 100 kPa: **a** axial strain–stress relation with $\Delta \epsilon _a = 0.01$ **b** axial strain–stress relation with $\Delta \epsilon _a = 0.03$

Moreover, the impact of stress correction algorithms and strain increment on model performance is validated through drained triaxial compression tests under varying loading conditions (strain increments). A one-step correction procedure is employed when stress violation causes the failure function larger than a tolerance of $10^{-3}$. Initially, a triaxial test is conducted under a constant loading rate ($\Delta \epsilon _a=0.01$) as shown in Fig. 5a. Note that the adopted strain increment is relatively large, thereby resulting in a larger peak strength compared to the reference due to integration error. When $e_0=1.08$, strain softening is not pronounced, and the evolution of the failure surface is relatively smooth. In this scenario, the stress state of the soil does not deviate significantly from the failure surface; and hence, the corrective effects are not apparent. However, for denser sand, strain softening becomes more evident, activating various stress correction algorithms. Among them, Method A leads to the most significant reduction in deviatoric stress; while, Method B approaches the results without correction, and Method C exhibits a moderate corrective effect. With increasing strain, deviatoric stresses obtained from different correction methods eventually converge to a critical state, validating the rationale behind employing these correction methods.

Subsequently, for a more comprehensive comparison of various correction methods, a scenario involving a sudden increase in loading is considered, which represents a situation frequently encountered in SPH simulations of large deformations. As depicted in Fig. 5b, the black curve serves as a reference, obtained through consistent and minor loading strain of $\Delta \epsilon _a = 0.003$. In the remaining four test sets, covering cases without correction, Method A, Method B, and Method C, the strain increments will be increased tenfold when the stress exceeds the failure threshold, i.e., $\epsilon _{a}=5\%$, resulting in a loading strain of $\Delta \epsilon _a = 0.03$. When modeling soil behavior within the framework of the hypoplastic model, the absence of stress correction results in the emergence of non-physical stress states that exceed the soil’s critical state. Conversely, the introduction of stress correction ensures computational stability, with the stress state gradually approaching the critical condition as the failure surface evolves. Consistent with the results in Fig. 5a, Method A appears to overcorrect the stress state of the soil; while, Method B does not yield satisfactory results and still exhibits significant deviations. Remarkably, Method C outperforms the other methods by consistently maintaining the stress state in proximity to the reference curve and steadily approaching it, thus demonstrating superior performance. As a result, Method C is selected for subsequent simulations to prevent any occurrence of stress drift.

### Simple shear test

**Fig. 6:**
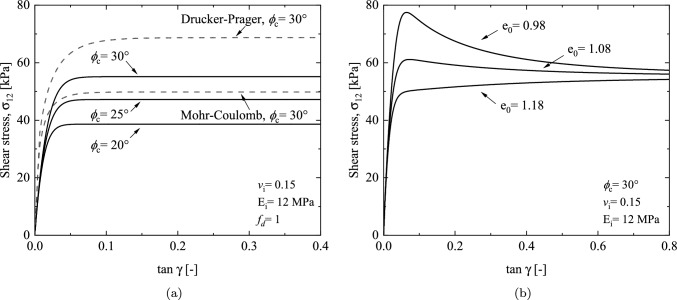
Numerical simulation of simple shear tests with $\sigma _3$= 100 kPa: **a** matched parameters of different constitutive models **b** influence of factor $f_d$ on shear stress

The performance of the proposed model is further validated through drained simple shear tests. Simple shear test holds particular relevance in predicting the shear-related behavior of soil. In the case of drained conditions, a constant vertical stress is maintained, thereby enabling continuous adjustment of the sample’s height and creating a condition similar to free drainage. Due to the fact that the factor $f_d$ does not affect the critical strength for the hypoplastic model (as shown in Figs. 4a and  6b), for simplicity, when comparing the shear strength between different constitutive models, $f_d$ is set to 1. The same parameters listed in Table 1 are used in the simulations.

The relations between the shear stress and the shear angle $\gamma$ using the parameters for the triaxial compression response and matched parameter for plane strain response of Drucker–Prager and Mohr–Coulomb models are presented in Fig. 6a, respectively. Although the three models demonstrate a good level of consistency in simulating the stress–strain relationships of triaxial drained tests, differences emerge when considering shear stress paths. Specifically, the Drucker–Prager criterion overestimates the shear strength (68.7 kPa), the Mohr–Coulomb criterion exhibits the most conservative prediction (49.8 kPa), and the hypoplastic model falls between the two (55.1 kPa). These variations are attributed to differences in the failure criteria utilized by each model. As depicted in Fig. 1a, under triaxial compression conditions, the failure surfaces of the three models intersect at a single point. However, for simple shear stress path, the radii of the hypoplastic failure surface locates between the Drucker–Prager and Mohr–Coulomb failure surfaces. In addition, the expansion and contraction of the hypoplastic failure surface can be controlled by adjusting the porosity. Figure 6b illustrates the phenomena of strain softening and strain hardening in sandy soil during the simple shear test. Due to the consideration of the critical state, the shear stress eventually converges to a consistent value of 55 kPa.

Up to this point, the capability of the simhypo-sand model for replicating essential soil mechanical behaviors, as well as the effectiveness of the stress correction algorithm, have been numerically validated. For validation of the simhypo-sand model against experimental data, the readers may refer to our previous work [43].

## Numerical simulation of large deformation problems

In this section, an in-house SPH code (modified from Liu and Liu [23]) is utilized to validate the performance of the hypoplastic model in large deformation problems. Two typical benchmarks are analyzed to evaluate the capabilities of the proposed model. Firstly, an investigation on a classical gravitational flow involving granular collapse is carried out. This case study aims to demonstrate the numerical stability and post-failure behavior of the hypoplastic model. Subsequently, a dynamic problem, namely penetration, commonly encountered in geotechnical engineering, will be conducted. By comparing the results with cone resistance data from centrifuge tests conducted by Bolton and Gui [3], the exceptional performance of the hypoplastic model in handling problems associated with substantial deformations and impact contact will be further elucidated. Finally, a real landslide deposition, the Tangjiashan landslide, is replicated to validate the efficacy of the hypoplastic model in disaster prediction. The model parameters (as shown in Table 2) used in the simulations are obtained through back analysis of previous studies [4, 15, 26].

**Table 2:** Parameters for SPH simulation in large deformation problems

Cases	$E_i$	$\nu _i$	$\phi _c$	$e_{\Gamma }$	$\lambda$	$\xi$	$\alpha$
Granular collapse	100.0 MPa	0.30	21.9^∘^	0.95	0.15	0.6	1.2
Penetration(MWG5)	22.4 MPa	0.33	30.0^∘^	0.82	0.12	0.6	1.5
Penetration(MWG10)	22.4 MPa	0.33	50.0^∘^	0.82	0.12	0.6	1.5
Tangjiashan landslide	10.0 MPa	0.20	20.0^∘^	0.95	0.15	0.6	1.2

### Granular collapse

In this simulation, a total of 3200 SPH particles are employed to create the initial rectangular soil domain, following the experimental setup as described by Nguyen et al. [26]. The sand parameters are determined through analysis of experiments by Nguyen et al. [26] and numerical simulations by Zhu et al. [53]. The domain dimensions measure 0.1 m in length and 0.2 m in width. The solid boundary conditions on the left and bottom sides align with the specifications presented by Bui et al. [5]. These boundary conditions are implemented using virtual particles to ensure both no-slip and free-slip effects. The inter-particle spacing, denoted as *d*, is set to 0.0025 m; while, the smoothing length is established as $h = 1.5d$. The coefficients of artificial viscosity, represented by $\alpha _\Pi$ and $\beta _\Pi$, are both set to 0.1. In accordance with the hypoplastic theory outlined in Sect. 2, the parameters for the granular material are determined by referencing the relevant properties of aluminum rods utilized in Nguyen’s experiments.

**Fig. 7:**
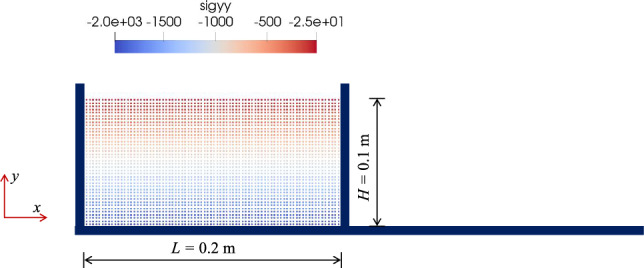
Initial state of the granular column

**Fig. 8:**
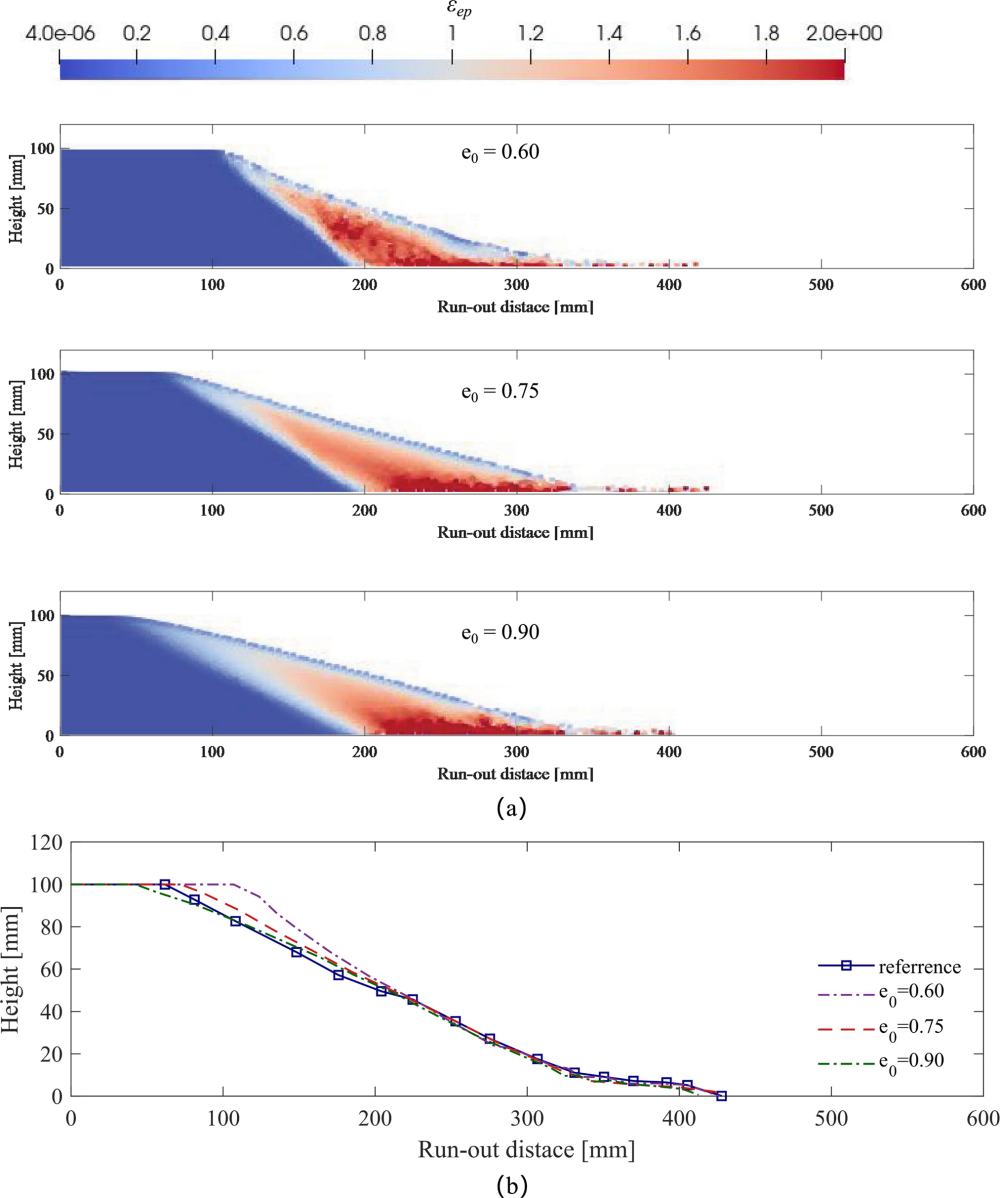
Failure profiles of granular column with different void ratio: **a** final deposition of granular flow **b** comparison of the free surface profiles

To effectively demonstrate the performance of the hypoplastic model under varying void ratios, a series of simulations are conducted. Three distinct initial void ratios 0.60, 0.75, and 0.90 are included, representing loose sand, medium-dense sand, and dense sand, respectively. Prior to soil failure, a vertical stress equilibrium is achieved under the influence of gravity through the use of a baffle, as depicted in Fig. 7. This resulted in a uniform distribution of vertical stress with increasing depth. Subsequently, in the simulation process, the baffle is removed to allow the soil to undergo failure. The final configurations of soil accumulations with different void ratios are shown in Fig. 8. With the same critical state, different cases exhibit similar soil failure profiles. Analyzing the equivalent plastic strain $\epsilon _{ep}=\sqrt{2(\epsilon _x^2+\epsilon _y^2+2\epsilon _{xy}^2)/3}$, the granular column can be partitioned into stable and unstable regions. A notable slip surface serves as the boundary, with the unstable region displaying higher plastic strain; while, the stable region remains static throughout the entire process.

**Fig. 9:**
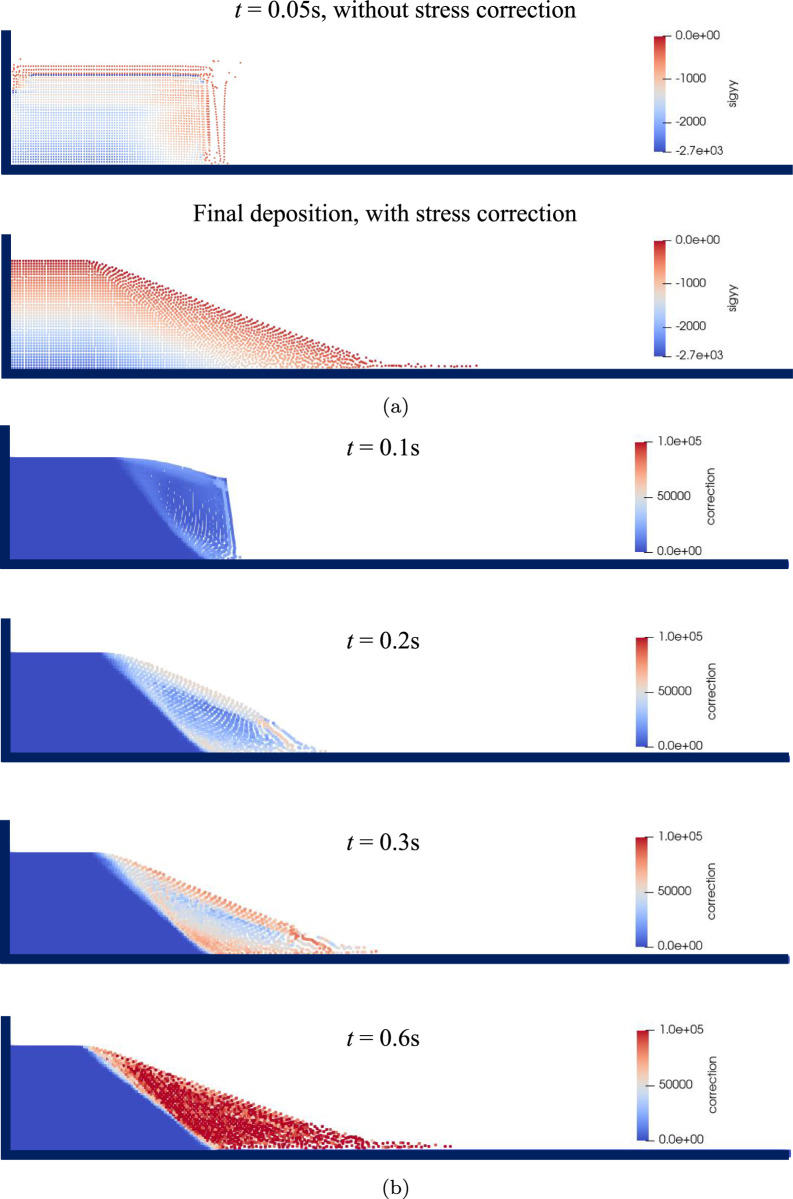
Collapse process of granular column ($e_0 = 0.75$) for simulation without and with stress correction treatment: **a** vertical stress distribution **b** the cumulative correction counts during the failure process

When comparing the experimental results with the three simulated soil surface distributions, it is evident that the final deposition of medium-dense sand with $e_0$ = 0.75 closely match the experimental data. When varying the void ratio, as observed in earlier element tests, dense sand has a higher peak strength. Consequently, in the $e_0$ = 0.60 case, the stable region of the soil is the largest (the purple curve has a longer platform segment). As anticipated, loose sand exhibits the most pronounced instability following shearing, with an shorter stable region falling short of the experimental result by approximately 19.2 mm. The difference is significantly greater when compared to dense sand, with a variation of 64.4 mm, exceeding the stable region of loose sand by approximately 151%. These distinctions underscore the paramount importance of the void ratio as a critical state parameter in accurately simulating the failure behavior of soil. Furthermore, the proposed hypoplastic model effectively captures the macroscopic post-failure characteristics of soil particles across varying void ratios.

To elucidate the significance of the stress correction algorithm in addressing large deformation challenges, simulations are executed using a case study with $e_0=0.75$. These simulations systematically compared scenarios incorporating and excluding the stress correction algorithm. Figure 9a compares the vertical stress distribution at the end of calculations with and without the stress correction. For the simulation without stress correction, some particles near the surface give rise to tensile stress state and abnormal stress magnitudes. This divergence in stress conditions results in anomalous accelerations, subsequently inducing a pathological velocity field. In contrast, the stress correction technique not only ensures the stability of the calculations but also yields a uniformly stress distribution. Figure 9b provide an overview of cumulative correction counts for each particle when employing the stress correction. In the stable region of the column, the correction count remains zero. However, in regions undergoing significant deformation, the correction count consistently surpasses $10^5$ by the end of this simulation. This underscores the pivotal role of the stress correction algorithm in effectively applying the hypoplastic model to large deformation simulations.

### Rigid penetrator in sand

To rigorously validate the efficiency of our proposed hypoplastic model for simulating cone penetration in sands of varying compaction, we have undertaken the replication of 70 g-centrifuge tests conducted by Bolton and Gui [3] at the renowned Cambridge Geotechnical Centrifuge Center. These specific tests involved cone penetration experiments in dry Fontainebleau sand, as further detailed in Table 3, utilizing a 10 mm diameter cone. For detailed information on the model preparation method and testing procedures, please refer to Bolton and Gui’s work [3, 4, 34].

**Table 3:** Properties of the Fontainebleau sand, referred from reference [3]

Grain diameter, $D_{50}$	0.181
Coefficient of uniformity, $C_u$	1.69
Maximum void ratio, $e_{max}$	0.92
Minimum void ratio, $e_{min}$	0.55
Specific gravity, $G_s$	2.65
Initial void ratio, $e_0$	0.59 (MWG5)/0.72 (MWG10)
Friction angle, $\phi$	50^∘^(MWG5)/30^∘^(MWG10)

**Fig. 10:**
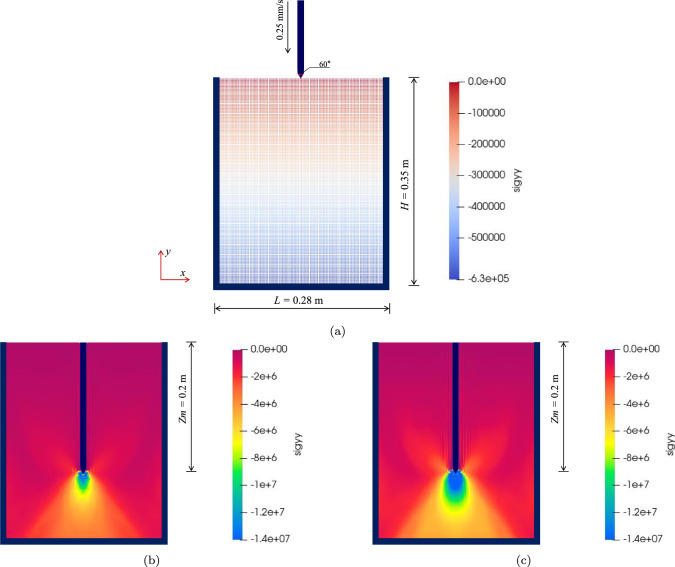
Vertical stress distribution in penetration simulation (MWG10): **a** initial profiles of penetration simulation **b**,**c** distribution of vertical stress in loose and dense sands

In this simulation, the primary aim of the SPH model is to reproduce the test results conducted by Bolton and Gui [3], with a specific emphasis on MWG5 (representing loose sand) and MWG9 (representing dense sand). These tests predominantly assess the resistance faced by the cone at various penetration depths, offering valuable insights into the practicality of the hypoplastic model for addressing geotechnical contact issues. The model dimensions are consistent with the finite element model used by Kouretzis [18], as depicted in Fig. 10a The soil is discretized into 15,680 discrete particles. Similar to granular collapse issue, the virtual particle method proposed by Bui et al. [5] is adopted to simulate no-slip boundaries at the bottom and sides. The inter-particle spacing is set to 0.0025 m; while, the smoothing length is established as $h = 1.5d$. The stress correction and artificial viscosity techniques are adopted here to ensure the stability of the simulations. The coefficients of artificial viscosity, $\alpha _\Pi$ and $\beta _\Pi$, are both set to 0.1.

The cone is represented by a rigid surface using the contact algorithm proposed by Wang et al. [39]. The equation for the contact algorithm between particle *i* and the rigid pile is illustrated as follows:

39 $$\begin{aligned} F_{n} = \frac{2m_i}{({\Delta t})^2}d_\text{intrusion}\varvec{n} \end{aligned}$$

40 $$\begin{aligned} F_{t} = {\left\{ \begin{array}{ll} \dfrac{2m_i}{(\Delta t)^2}\Delta u_t\varvec{\tau } &{} \text {if } |F_t| \le \mu _f |F_n|, \\ \mu _f F_n\varvec{\tau } &{} \text {otherwise.} \end{array}\right. } \end{aligned}$$

where $d_{\text {intrusion}}$ is the intrusive distance of particle *i*, $\varvec{n}$ and $\varvec{\tau }$ represent the normal and tangent vectors of the rigid surface, respectively. $\Delta u_t$ is the relative displacement of particle *i* and the rigid surface, and the friction coefficient $\mu _f$ is determined through Kouretzis’ back analysis, set to 0.5. For the sake of consistency with Kouretzis [4, 34], results are presented by contrasting cone resistance ($q_c$) with the corrected prototype penetration depth ($z_{pc}$):

41 $$\begin{aligned} z_{pc} = z_p \left[1+\frac{z_m}{2R_m}\right],\quad z_p=Nz_m \end{aligned}$$

where $z_m$ is the model penetration depth, $R_m = 0.3755$ m is the average radius to the surface of the sand specimen measured from the central axis of the centrifuge rig, and $N = 70$ is test acceleration level.

**Fig. 11:**
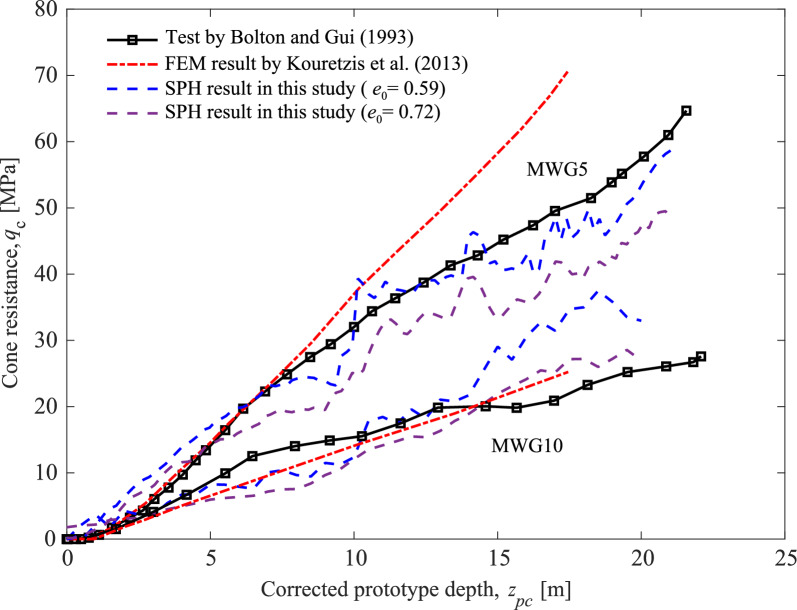
Curve of cone resistance versus depth

In this simulation, void ratios of 0.59 and 0.72 are employed to characterize dense sand and loose sand, respectively, corresponding to two centrifuge tests, MWG5 and MWG10. Prior to penetration, the initial ground stress equilibrium is established. Subsequently, a rigid cone descends vertically at a controlled speed of 0.25 mm/s, reaching a final penetration depth denoted as $z_m$ at 0.20 m. Figures 10b and 10c illustrate the post-penetration vertical stress distribution in loose and dense sands in test MWG10. Notably, a pronounced stress concentration is observed at the pile head position in cloud chart in Figs. 10b and c, with a larger vertical compressive stress distribution in dense sand compared to loose sand.

For consistency with Kouretzis’ work [18], the results are presented by contrasting cone resistance ($q_c$) with the corrected prototype penetration depth ($z_{pc}$) in Fig. 11. Upon scrutinizing the corrected core resistance-depth curves, it becomes evident that, due to the incorporation of porosity effects, the variation in core resistance is not strictly linear. When compared to Kouretzis’ elastoplastic constitutive modeling, the hypoplastic model demonstrates a closer alignment with experimental data. Concurrently, the heightened peak strength of dense sand is more intuitively reflected in core resistance, with the core resistance in dense sand surpassing that in loose sand by approximately 8.9 MPa in test MWG10, equivalent to around 32.1%, and 9.5 MPa in test MWG5, equivalent to around 19.2%.

### Tangjiashan landslide

In the realm of landslide research, numerous scholars have undertaken the replication of specific events through the application of SPH coupled with various constitutive models grounded in fluid mechanics, such as the Bingham flow model and equivalent Newtonian viscosity model [1, 12, 14]. Although these models have exhibited commendable efficacy in forecasting the ultimate deposition of sliding masses, it is imperative to acknowledge that the representation of soil behavior within these frameworks is inherently simplified. These simplifications notably pertain to the treatment of nonlinear behavior, failure mechanics, and the stress–strain relationship. This section endeavors to overcome these limitations by introducing the proposed hypoplastic model. Focusing on a real case study-the Tangjiashan landslide in Sichuan, China-the study meticulously reproduces the event, aiming to underscore the hypoplastic model’s ability to emulate real-world scenarios.

**Fig. 12:**
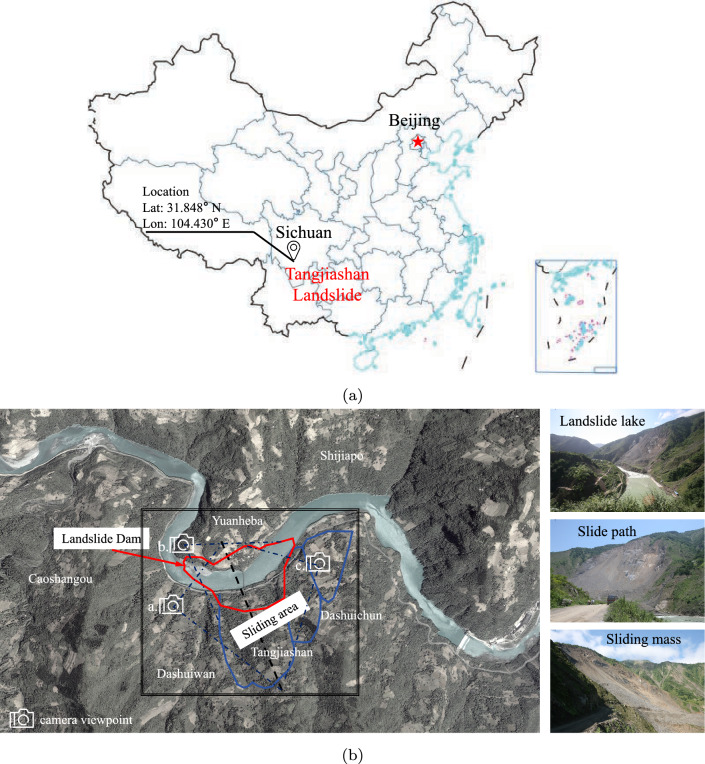
Illustration of the Tangjiashan landslide: **a** landslide location and **b** on-site images

The “5.12" Wenchuan earthquake triggered a series of geological hazards, including collapses and landslides, leading to the formation of 104 landslide dam-induced barrier lakes up to August 22, 2008 [13]. Among these hazards, the Tangjiashan landslide dam (Fig. 12a, situated on the right bank of the Tongkou River approximately 6 km upstream from Beichuan County, China, stands out as the largest in terms of blockage scale, potential hazards, and susceptibility to secondary disasters. Preceding the occurrence of the landslide, Tangjiashan’s terrain exhibited a steep slope of approximately 40^∘^, with a base elevation of about 665 m and the watershed ridge reaching nearly 1500 m, resulting in a significant relative elevation difference of 835 m. This steep topography contributed to a high-speed landslide, obstructing the river and forming a barrier dam with dimensions, including a downstream length of 803.4 m and a maximum width across the river of 611.8 m [7], as shown in Fig. 12b.

**Fig. 13:**
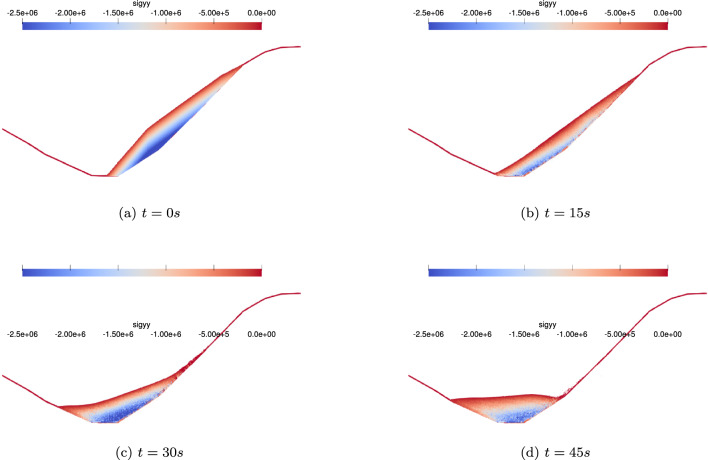
Deposition process of the Tangjiashan landslide in SPH simulation

**Fig. 14:**
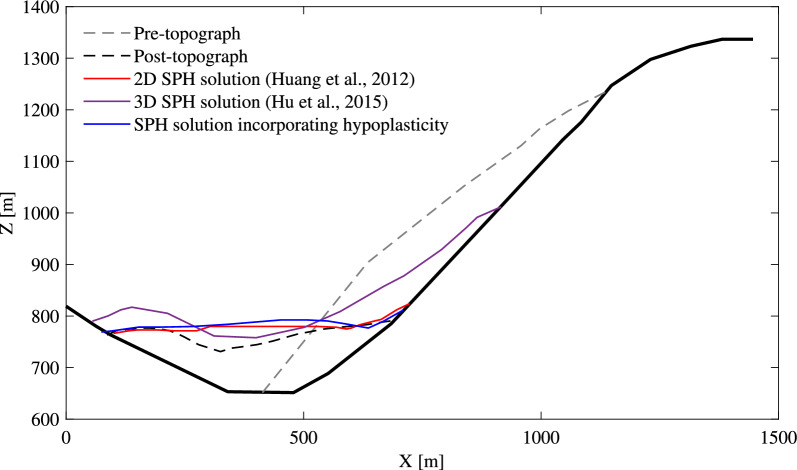
Pre- and post-topographies of the Tangjiashan landslide

In the previous simulation of the Tangjiashan landslide, Huang and Dai [14], and Hu et al. [12] correlated the yield strength of the Bingham fluid with the Mohr–Coulomb failure criterion, providing the initial shear strength for the soil. They assigned values of a friction angle of 30^∘^ and cohesion of 30 kPa. However, these parameters were too strong for soil mechanics considerations; and therefore, their simulation parameters are not adopted in this study. Through post analysis, the friction angle is determined to be 20^∘^, consistent with Bao et al.’s study [1]; while, cohesion was not considered. The initial void ratio $e_0$ is set to 0.7, and the remaining parameters are listed in Table 2. The sliding mass is discretized into 16,476 discrete particles, and the inter-particle spacing is uniformly set to 2 m. To mitigate soil particle penetration, the sliding surface incorporates a densely arranged configuration of virtual particles, as proposed by Bui et al. [5]. At the bottom, the particle spacing is specifically fixed at 1 m. Vertically, the sliding surface is organized into four layers, totaling 8,586 particles. The smoothing length is established as $h = 1.5d$. Similarly, artificial viscosity and stress correction are used to ensure the stability of the simulations. The coefficients of artificial viscosity, $\alpha _\Pi$ and $\beta _\Pi$, are set to 0.25 and 0.1, respectively.

Figure 13 illustrates the simulated run-out process of the landslide and the evolution of the sliding mass’s topography during the simulation. The SPH numerical model effectively captures the complete landslide processes of geomaterials. In addition to illustrating slope configurations, the SPH analysis reveals crucial dynamic behaviors, including impact force and flow velocity, which are essential for analyzing the run-out of landslide-like flows. These dynamic insights can be leveraged for hazard assessments, providing valuable information to decision-makers aiming to enhance disaster prevention measures. Note that in simulations with extreme high strain rate, there are still occurrences of stress noise. Further research is needed to improve the accuracy of the time integration scheme [42] or to implement stress smoothing algorithms [31].

However, on a practical level, capturing real-time impact force and velocity during landslides in mountainous areas proves to be a considerable challenge. Therefore, this study relies on a comparison of pre- and post-earthquake terrain data to validate the effectiveness of proposed hypoplastic model. As depicted in Fig. 14, the predicted post-deposition of the sliding mass aligns closely with the actual post-topography observed in the field, as well as with findings from previous 2D simulations [12, 15]. This noteworthy similarity underscores the importance of integrating the hypoplastic model with SPH method for robust disaster prevention and mitigation strategies.

## Conclusions

This study introduces the simhypo-sand model, a simple hypoplastic constitutive model for granular material. It derives the explicit expression of its failure surface, elucidates the physical interpretation of its individual parameters, and outlines their determination method. Additionally, a stress correction algorithm is incorporated to ensure the operational stability of the model, especially in problems involving large deformations. Subsequently, the practical applicability and robustness of the model are validated through several elementary tests and large deformation problems. The primary conclusions of this study are as follows:

1. The hypoplastic model holds significant practical value due to its utilization of only 7 parameters to capture salient soil constitutive relationships. In comparison with ideal elastoplastic models with Mohr–Coulomb and Drucker–Prager failure criteria, it effectively simulates the nonlinear response of soils. Furthermore, by introducing the critical state concept, the model aptly reflects the evolution of failure surfaces, enabling accurate modeling of strain hardening or strain softening behavior in sand under varying initial conditions.
2. In the model’s implementation, three stress correction methods, as proposed by Potts and Gens [32], are integrated and compared. Among these methods, Method C, i.e., projecting back by reducing $J_2$ at constant $I_{1}$, demonstrates the most favorable impact, efficiently correcting stress states without compromising soil peak strength and preventing any occurrence of stress drift.
3. Regarding the numerical implementation of the model, the stress–strain curves of the hypoplastic model are compared against those of the traditional Mohr–Coulomb and Drucker–Prager models through elementary tests. The proposed failure function, which varies with the shape and changes in void ratio, highlighted the model’s capability to accurately simulate soil constitutive relationships across varying stress paths.
4. In the context of large deformation scenarios, including granular collapse flow, rigid penetration, and landslide run-out, the model demonstrated excellent agreement with experimental data and monitoring data. Furthermore, it revealed distinct behaviors between loose and dense sands. Dense sands exhibited elevated peak shear strength, leading to diminished sliding distances and augmented cone resistance in comparison with loose sands. Notably, the model’s effectiveness in simulating granular collapse flow underscores its clear suitability for practical applications in predicting landslide and debris flow hazards.

## Data Availability

All the data and test programs of this study are available from the corresponding author upon reasonable request.

## Appendix A. Simhypo-sand model

The concrete formulation of the simhypo-sand model is given as:

A.1 $$\begin{aligned} \mathring{\varvec{\sigma }}=f_s\Bigg [\textrm{tr}(\varvec{\sigma })\dot{\varvec{\epsilon }}+ f_v\textrm{tr}(\dot{\varvec{\epsilon }})\varvec{\sigma }+ a^2\frac{\textrm{tr}(\varvec{\sigma }\dot{\varvec{\epsilon }})}{\textrm{tr}(\varvec{\sigma })}\varvec{\sigma }+ f_da(\varvec{\sigma }+\varvec{\sigma }^{*})\Vert \dot{\varvec{\epsilon }}\Vert \Bigg ] \end{aligned}$$

where $f_s$ and $f_v$ are multiplier accounting for the stiffness and volumetric response of the materials, respectively, as follows:

A.2 $$\begin{aligned} f_s= -\frac{E_i}{3(1+\nu _i)\sigma _c},\; f_v= \frac{3\nu _i+f_da\sqrt{1+2\nu _i^2}}{1-2\nu _i} -\frac{a^2}{3} \end{aligned}$$

in which $E_i$ and $\nu _i$ are material parameters. $\sigma _c =100$ kPa is used for material calibration.

The density factor $f_d$ is given as follows:

A.3 $$\begin{aligned} f_d=\left( \frac{e}{e_c}\right) ^\alpha \end{aligned}$$

in which $\alpha$ is a material parameter, *e* is the current void ratio; $e_c$ is the critical state void ratio, which is dependent on the stress level, as follows:

A.4 $$\begin{aligned} e_c=e_\Gamma -\lambda \left( \frac{p^\prime }{p_\text {a}}\right) ^\xi \end{aligned}$$

where $e_\Gamma$, $\lambda$, and $\xi$ are material parameters for characterizing the critical state of granular materials. $p^\prime$ is the effective mean stress and $p_\text {a}$ = $-$101.325 kPa is the atmospheric pressure for normalization.

In this model, the factor *a* regulates the strength of the material through:

A.5 $$\begin{aligned} a= & {} \frac{\eta \sqrt{3}(3-\text {sin}\phi _c)}{2\sqrt{2}\text {sin}\phi _c}\; \text {with } \;\nonumber \\ \eta= & {} \frac{2I_1}{3\sqrt{I_1^2-3I_2}\sqrt{(I_1I_2-I_3)/(I_1I_2-9I_3)-1}} \end{aligned}$$

where $\phi _c$ is the critical state friction angle and the factor $\eta$ is adopted to incorporate the SMP failure criterion.

The model contains 7 material parameters that can be classified into two groups: (1) Four parameters for the hypoplastic model: $E_i$, $\nu _i$, $\phi _c$, and $\alpha$; (2) Three parameters for the critical state of granular materials: $e_\Gamma$, $\lambda$, and $\xi$. All the parameters can be obtained from triaxial compression tests. The three material parameters of the hypoplastic model $E_i$ (initial elastic modulus), $\nu _i$ (initial Poisson’s ratio), and $\phi _c$ (critical state friction angle) can be readily attained from a single triaxial compression test with confining pressure of 100 kPa. $\alpha$ can be determined by trial and error based on the density effect on strain softening or hardening. The critical state parameters $e_{\Gamma }$, $\lambda$, and $\xi$ can be measured from an $e-\text {log}(p')$ plot of critical states based on three triaxial compression tests. In addition, the constitutive model requires the initial value of void ratio.

## Appendix B. Explicit scheme for Method C

Equation (B.1), as modified from Wang et al.’s work [42], elucidates the stress correction Method C embedded within the Drucker–Prager model framework. This method for proposed hypoplastic model is expressed as follows:

B.1 $$\begin{aligned} \text {Method C:} \quad \varvec{\sigma }_{\text {correction}}^{*}=\alpha _{c}\varvec{\sigma }_\text {trial}^{*},\quad I_{1(\text {correction})}=I_{1(\text {trial})} \end{aligned}$$

where $\alpha _{c}$ serves as the correction factor associated with the trial values of $J_2$ and $I_1$ and is defined by:

B.2 $$\begin{aligned} \alpha _{c}=-\dfrac{\eta I_1}{\sqrt{J_2^{\text {trial}}}} \end{aligned}$$

the scale multiplier $\eta$ is determined as:

B.3 $$\begin{aligned} \eta =\sqrt{\dfrac{2A_1}{-A_2-\sqrt{A_2^2-4A_1A_3}}} \end{aligned}$$

with the components $A_1$, $A_2$, and $A_3$ given by:

B.4 $$\begin{aligned} A_1=\dfrac{a^2f_d^2}{3}-(1+f_v+\dfrac{a^2}{3})^2 \end{aligned}$$

B.5 $$\begin{aligned} A_2=2f_d^2a^2((1+f_v+\dfrac{a^2}{3})^2+2f_v+3)-4a^2(1+f_v+\dfrac{a^2}{3}) \end{aligned}$$

B.6 $$\begin{aligned} A_3=\dfrac{4}{3}f_d^2a^6-4a^4 \end{aligned}$$

## References

[1] Bao Y, Huang Y, Liu G, Wang G (2020) SPH simulation of high-volume rapid landslides triggered by earthquakes based on a unified constitutive model Part I: initiation process and slope failure. Int J Comput Methods 17(04):1850150. 10.1142/S0219876218501505 10.1142/S0219876218501505

[2] Bauer E (1996) Calibration of a comprehensive hypoplastic model for granular materials. Soils Found 36(1):13–26. 10.3208/sandf.36.13 10.3208/sandf.36.13

[3] Bolton MD, Gui M (1993) The study of relative density and boundary effects for cone penetration tests in centrifuge. Cambridge University Engineering Department, CUED/D-SOILS/TR256: UK

[4] Bolton MD, Gui MW, Garnier JF, Corte J (1999) Centrifuge cone penetration tests in sand. Géotechnique 49(4):543–552. 10.1680/geot.1999.49.4.543 10.1680/geot.1999.49.4.543

[5] Bui HH, Fukagawa R, Sako K, Ohno S (2008) Lagrangian meshfree particles method (SPH) for large deformation and failure flows of geomaterial using elastic-plastic soil constitutive model. Int J Numer Anal Meth Geomech 32(12):1537–1570. 10.1002/nag.688 10.1002/nag.688

[6] Chen JY, Peng C, Lien FS (2019) Simulations for three-dimensional landmine detonation using the SPH method. Int J Impact Eng 126:40–49. 10.1016/j.ijimpeng.2018.12.004 10.1016/j.ijimpeng.2018.12.004

[7] Cui P, Zhu YY, Han YS, Chen XQ, Zhuang JQ (2009) The 12 May Wenchuan earthquake-induced landslide lakes: distribution and preliminary risk evaluation. Landslides 6:209–223. 10.1007/s10346-009-0160-9 10.1007/s10346-009-0160-9

[8] Dafalias YF, Herrmann L (1986) Bounding surface plasticity. II: application to isotropic cohesive soils. J Eng Mech 112(12):1263–1291. 10.1061/(ASCE)0733-9399 10.1061/(ASCE)0733-9399

[9] Gudehus G (1996) A comprehensive constitutive equation for granular materials. Soils Found 112(12):1263–1291. 10.3208/sandf.36.1 10.3208/sandf.36.1

[10] He YQ, Liao HJ, Wu W, Wang S (2023) Hypoplastic modelling of inherent anisotropy in normally and overconsolidated clays. Acta Geotech 18:6315–6333. 10.1007/s11440-023-01923-3 10.1007/s11440-023-01923-3

[11] He YQ, Wang S, Liao HJ, Wu W (2022) A hypoplastic constitutive model for structured soils. Comput Geotech 151(3):104935. 10.1016/j.compgeo.2022.104935 10.1016/j.compgeo.2022.104935

[12] Hu M, Liu MB, Xie MW, Liu GR (2015) Three-dimensional run-out analysis and prediction of flow-like landslides using smoothed particle hydrodynamics. Environ Earth Sci 73:1629–1640. 10.1007/s12665-014-3513-1 10.1007/s12665-014-3513-1

[13] Hu XW, Huang RQ, Shi YB, Lu XP, Zhu HY, Wang XR (2009) Analysis of blocking river mechanism of Tangjiashan landslide and dam-breaking mode of its barrier dam. Chin J Rock Mechan Eng 28(1):181–189

[14] Huang Y, Dai Z (2014) Large deformation and failure simulations for geo-disasters using smoothed particle hydrodynamics method. Eng Geol 168:86–97. 10.1016/j.enggeo.2013.10.022 10.1016/j.enggeo.2013.10.022

[15] Huang Y, Zhang W, Xu Q, Xie P, Hao L (2012) Run-out analysis of flow-like landslides triggered by the Ms 8.0 2008 Wenchuan earthquake using smoothed particle hydrodynamics. Landslides 9:275–283. 10.1007/s10346-011-0285-5 10.1007/s10346-011-0285-5

[16] Jiao H, Lv Y, Chen D, Huang W, Su Y (2024) Numerical implementation of the hypoplastic model for SPH analysis of soil structure development in extremely large deformation. Comput Geotech 166:106014. 10.1016/j.compgeo.2023.106014 10.1016/j.compgeo.2023.106014

[17] Kolymbas D, Herle I, Von Wolffersdorff PA (1995) Hypoplastic constitutive equation with internal variables. Int J Numer Anal Meth Geomech 19(6):415–436. 10.1002/nag.1610190604 10.1002/nag.1610190604

[18] Kouretzis GP, Sheng D, Wang D (2014) Numerical simulation of cone penetration testing using a new critical state constitutive model for sand. Comput Geotech 56:50–60. 10.1016/j.compgeo.2013.11.002 10.1016/j.compgeo.2013.11.002

[19] Li XS, Wang Y (1998) Linear representation of steady-state line for sand. J Geotech Geoenviron Eng 124(12):1215–1217. 10.1061/(ASCE)1090-0241 10.1061/(ASCE)1090-0241

[20] Liao D, Yang Z, Wang S, Wu W (2022) Hypoplastic model with fabric change effect and semifluidized state for post-liquefaction cyclic behavior of sand. Int J Numer Anal Meth Geomech 46(17):3154–3177. 10.1002/nag.3444 10.1002/nag.3444

[21] Liao D, Yang Z, Wang S, Wu W (2023) A hypoplastic model for cemented sand under monotonic and cyclic loading. Can Geotech J. 10.1139/cgj-2023-0079 10.1139/cgj-2023-0079

[22] Liao D, Wang S, Zhang C (2024) A hypoplastic model for crushable sand under a wide range of stress levels. Acta Geotech. 10.1007/s11440-024-02230-1 10.1007/s11440-024-02230-1

[23] Liu GR, Liu MB (2003) Smoothed particle hydrodynamics: a meshfree particle method. World Scientific Publishing, Singapore. 10.1142/5340

[24] Mašín D (2005) A hypoplastic constitutive model for clays. Int J Numer Anal Meth Geomech 29(4):311–336. 10.1002/nag.416 10.1002/nag.416

[25] Monaghan JJ (1994) Simulating free surface flows with SPH. J Comput Phys 110(2):399–406. 10.1006/jcph.1994.1034 10.1006/jcph.1994.1034

[26] Nguyen CT, Bui HH, Fukagawa R (2015) Failure mechanism of true 2D granular flows. J Chem Eng Jpn 48(6):395–402. 10.1252/jcej.14we358 10.1252/jcej.14we358

[27] Pastor M, Haddad B, Sorbino G, Cuomo S, Drempetic V (2009) A depth-integrated, coupled SPH model for flow-like landslides and related phenomena. Int J Numer Anal Meth Geomech 33(2):143–172. 10.1002/nag.705 10.1002/nag.705

[28] Pastor M, Zienkiewicz OC, Chan AHC (1990) Generalized plasticity and the modelling of soil behavior. Int J Numer Anal Meth Geomech 14(3):151–190. 10.1002/nag.1610140302 10.1002/nag.1610140302

[29] Peng C, Wu W, Yu H, Wang C (2015) A SPH approach for large deformation analysis with hypoplastic constitutive model. Acta Geotech 10:703–717. 10.1007/s11440-015-0399-3 10.1007/s11440-015-0399-3

[30] Peng C, Guo X, Wu W, Wang Y (2016) Unified modelling of granular media with smoothed particle hydrodynamics. Acta Geotech 11:1231–1247. 10.1007/s11440-016-0496-y 10.1007/s11440-016-0496-y

[31] Peng C, Wang S, Wu W, Yu H, Wang C, Chen J (2019) LOQUAT: an open-source GPU-accelerated SPH solver for geotechnical modeling. Acta Geotech 14:1269–1287. 10.1007/s11440-019-00839-1 10.1007/s11440-019-00839-1

[32] Potts DM, Gens A (1985) A critical assessment of methods of correcting for drift from the yield surface in elasto-plastic finite element analysis. Int J Numer Anal Meth Geomech 9(2):149–159. 10.1002/nag.1610090204 10.1002/nag.1610090204

[33] Poulos SJ (1981) The steady state of deformation. J Geotech Eng Div 107(5):553–562. 10.1061/AJGEB6.0001129 10.1061/AJGEB6.0001129

[34] Schofield AN (1980) Cambridge geotechnical centrifuge operations. Geotechnique 30(3):227–268. 10.1680/geot.1980.30.3.227 10.1680/geot.1980.30.3.227

[35] Sloan SW (1987) Substepping schemes for the numerical integration of elastoplastic stress-strain relations. Int J Numer Meth Eng 24(5):893–911. 10.1002/nme.1620240505 10.1002/nme.1620240505

[36] Soleimani M, Weißenfels C (2021) Numerical simulation of pile installations in a hypoplastic framework using an SPH based method. Comput Geotech 104006:133. 10.1016/j.compgeo.2021.104006 10.1016/j.compgeo.2021.104006

[37] Von Wolffersdorff PA (1996) A hypoplastic relation for granular materials with a predefined limit state surface. Mech Cohesive-frictional Mater Int J Exper Model Comput Mater Struct 1(3):251–271. 10.1002/(SICI)1099-1484 10.1002/(SICI)1099-1484

[38] Wallin M, Ristinmaa M, Ottosen NS (2003) Kinematic hardening in large strain plasticity. Eur J Mech-A/Solids 22(3):341–356. 10.1016/S0997-7538(03)00026-3 10.1016/S0997-7538(03)00026-3

[39] Wang J, Chan D (2014) Frictional contact algorithms in SPH for the simulation of soil-structure interaction. Int J Numer Anal Meth Geomech 38(7):747–770. 10.1002/nag.2233 10.1002/nag.2233

[40] Wang S, Wu W (2021) Validation of a simple model for overconsolidated clay. Acta Geotech 16:31–41. 10.1007/s11440-020-01105-5 10.1007/s11440-020-01105-5

[41] Wang S, Wu W (2021) A simple hypoplastic model for overconsolidated clays. Acta Geotech 16:21–29. 10.1007/s11440-020-01000-z 10.1007/s11440-020-01000-z

[42] Wang S, Wu W, Peng C, He XZ, Cui DS (2018) Numerical integration and FE implementation of a hypoplastic constitutive model. Acta Geotech 13:1265–1281. 10.1007/s11440-018-0684-z 10.1007/s11440-018-0684-z

[43] Wang S, Wu W, Yin ZY, Peng C, He XZ (2018) Modelling the time-dependent behaviour of granular material with hypoplasticity. Int J Numer Anal Meth Geomech 42(12):1331–1345. 10.1002/nag.2793 10.1002/nag.2793

[44] Wei X, Yang J (2019) A critical state constitutive model for clean and silty sand. Acta Geotech 14:329–345. 10.1007/s11440-018-0675-0 10.1007/s11440-018-0675-0

[45] Woo S, Seo H, Kim J (2017) Critical-state-based Mohr–Coulomb plasticity model for sands. Comput Geotech 92:179–185. 10.1016/j.compgeo.2017.08.00 10.1016/j.compgeo.2017.08.00

[46] Wu W, Bauer E (1994) A simple hypoplastic constitutive model for sand. Int J Numer Anal Meth Geomech 18(12):833–862. 10.1002/nag.1610181203 10.1002/nag.1610181203

[47] Wu W, Bauer E, Kolymbas D (1996) Hypoplastic constitutive model with critical state for granular materials. Mech Mater 23(1):45–69. 10.1016/0167-6636(96)00006-3 10.1016/0167-6636(96)00006-3

[48] Wu W, Lin J, Wang XT (2017) A basic hypoplastic constitutive model for sand. Acta Geotech 12:1373–1382. 10.1007/s11440-017-0550-4 10.1007/s11440-017-0550-4

[49] Xu G, Wu W, Qi J (2016) Modeling the viscous behavior of frozen soil with hypoplasticity. Int J Numer Anal Meth Geomech 40(15):2061–2075. 10.1002/nag.2516 10.1002/nag.2516

[50] Yao Y, Lu D, Zhou A, Zou B (2016) Generalized non-linear strength theory and transformed stress space. Sci China Ser E: Technol Sci 47:691–709. 10.1360/04ye0199 10.1360/04ye0199

[51] Zhan L, Peng C, Zhang B, Wu W (2019) Three-dimensional modeling of granular flow impact on rigid and deformable structures. Comput Geotech 112:257–271. 10.1016/j.compgeo.2019.03.019 10.1016/j.compgeo.2019.03.019

[52] Zhao Y, Jin W, Klinger J, Dayton DC, Dai S (2023) SPH modeling of biomass granular flow: theoretical implementation and experimental validation. Powder Technol 426:118625. 10.1016/j.powtec.2023.118625 10.1016/j.powtec.2023.118625

[53] Zhu CW, Wu W, Peng C, Wang S, Wei X (2024) SPH implementation of a critical state-based hypoplastic model for granular materials in large-deformation problems. Comput Geotech 166:106011. 10.1016/j.compgeo.2023.106011 10.1016/j.compgeo.2023.106011

[54] Zhu H, Martys NS, Ferraris C, De Kee D (2010) A numerical study of the flow of Bingham-like fluids in two-dimensional vane and cylinder rheometers using a smoothed particle hydrodynamics (SPH) based method. J Non Newton Fluid Mech 165(7–8):362–375. 10.1016/j.jnnfm.2010.01.012 10.1016/j.jnnfm.2010.01.012

